# The Christmas Season and the Dying Year

**Published:** 1899-12-30

**Authors:** 


### The Hospital

A Journal of

The Medical Sciences and Hospital Administration.

Vol. XXVII.—No. 692.] EDITED BY SIR HENRY BURDETT, K.C.B., AND SOLOMON C. SMITH, M.D., M.R.C.P. [December 30, 1899.

### THE CHRISTMAS SEASON AND THE DYING YEAR.

It is one of the privileges of great genius to furnish landmarks, milestones, as it were, upon the road of Time, by which not only may the wayfarer estimate his progress, but from which the historian may obtain materials for his conclusions. Scarcely more than fifty years have passed away since those who were then young or middle-aged were annually delighted by the recurring Christmas books of Dickens and Thackeray, by "The Chimes," "The Cricket on the Hearth," "Our Street," "Mrs. Perkins's Ball," and others of a similar character, depicting phases of civilisation which are now as extinct as the dodo, and recalling a time when morbid introspection and self-analysis were the amusements of hysterical girls and the occupations of fantastic devotees, instead of being the favourite themes of the most popular novelists. By many who are not very far advanced upon the downward slope of life the Christmas themes and topics of fifty years ago are as well remembered as if they were of yesterday; and the contrasts which they present to those of the present time are many and curious. The galvanised corpse of the Christmas of 1849 does, indeed, haunt a few of our principal thoroughfares, in the shape of advertisements displaying a white-headed and white-bearded old gentleman in scarlet robes, crowned with holly or with mistletoe, and with his hands overflowing with gifts; or in the shape of the name of Santa Claus as part of the programme of an entertainment. Beyond these limits, the old traditions scarcely survive; and those who are still young will find it scarcely possible to realise what was once their vividness and their force. Even so lately as at the period of which we are speaking, the Christmas season was almost the only time for family reunions; events always pleasant in prospect, although the felicity of realisation may leave something to be desired; and it thus became specially a time of hospitality, with its natural concomitant of feasting. The geniality of the hearth was heightened by its contrast with the frequent inclemency of the season, and the difficulties incidental to locomotion gave zest to the time when they had been overcome. The children of those days were boys and girls, easily pleased by amusements or entertainments, and wholesomely ready for a game at romps. To-day, thanks to railways and to steamboats, to Cook and to Gaze, family reunions are limited to no season, and occur whenever the persons composing them have any desire to come together, while the effect of physiological inquiries into the principles governing nutrition has not been favourable to the overmuch consumption of good cheer. The difficulties of locomotion have dwindled to the inconvenience of a train three hours late, together with the occasional loss of a parcel or of a baby. Children have become little men and women, mostly overdressed, and overconscious of the overdressing; and they examine with critical eyes the provision made for amusing them. "Is that all?" was asked last year by a young lady of eight, at the close of a "juvenile party," about which no money had been spared. The only real survival of the ancient spirit which has fallen under our observation has been furnished by another young lady of the same age, who gravely objected to go away with her parents for the week end, because, she said, "Santa Claus would not know her address."

In many ways, no doubt, the changes have been for the better. Fifty years ago the system of domestic management was based almost entirely upon credit, as it is now based almost entirely upon cash. At Christmas the unhappy paterfamilias was overwhelmed with bills from all quarters, melancholy reminders of forgotten indulgences; and the season of supposed joviality was often clouded by considerations as to how both ends could be made to meet. The most remarkable feature of the establishment of one of the Dickens heroes was that all the bills were paid before midnight on the last day of the year; whereas now, in a similar establishment, there would presumably be no bills to pay, unless, indeed, it were that of the doctor, who is now almost the sole representative of a once universal custom. Another great advance has been in the diminution of alcoholic excess, a diminution happily coincident with an increased national consumption of alcoholic liquor, and thus displaying at once an appreciation of its use as distinguished from its abuse, and a higher standard of domestic comfort and enjoyment. The turning-point of these changes, in all probability, the opening of the gulf which separates the Christmas of the forties from the Christmas of to-day, must be assigned to that of the year 1854, when, as now, the great festival coincided with a period of profound national gloom and anxiety. Then, as now, the bravest troops in the world had been hurled into war for which no adequate preparation had been made by the authorities at home. In those days they were sacrificed to fever, to starvation, to frostbite, to scurvy; as they have now been sacrificed to official unwillingness to believe in the strength and matured preparedness of the enemy, and to the inadequacy of an artillery which has not been kept up to the standard of the day. Then, as now, many hearths were desolate, and many hearts torn with anxiety for those most dear to them. May the parallel be complete; and, now as then, may the indomitable resolution of the country be sufficient to overcome the obstacles to success which have been permitted to accumulate by the blindness of its rulers.

